# Comparing gender awareness in Dutch and Swedish first-year medical students - results from a questionaire

**DOI:** 10.1186/1472-6920-12-3

**Published:** 2012-01-12

**Authors:** Jenny Andersson, Petra Verdonk, Eva E Johansson, Toine Lagro-Janssen, Katarina Hamberg

**Affiliations:** 1 Department of Public Health and Clinical Medicine, Family Medicine, Umeå University, 901 85 Umeå, Sweden and National Graduate School of Gender Studies, Umeå University, 901 87 Umeå, Sweden; 2 VU University Medical Center, Department of Medical Humanities, EMGO Institute for Health and Care Research, van der Boechorsstraat 7, 1081 BT Amsterdam, the Netherlands; 3 Department of Public Health and Clinical Medicine, Family Medicine, Umeå University, 901 85 Umeå, Sweden; 4 Department of Primary and Community Care, Centre for Family Medicine, Geriatric and Public Health, Unit Women's Studies, Radboud University Nijmegen Medical Centre, P.O. Box 9101, 6500 HB Nijmegen, The Netherlands

**Keywords:** Medical curricula, medical students, gender implementation, gender, attitudes, stereotypical thinking

## Abstract

**Background:**

To ascertain good and appropriate healthcare for both women and men implementation of gender perspectives in medical education is needed. For a successful implementation, knowledge about students' attitudes and beliefs about men, women, and gender is crucial. The aim of this study was to compare attitudes to gender and gender stereotyping among Dutch and Swedish male and female medical students.

**Methods:**

In this cross-sectional study, we measured the attitudes and assumptions about gender among 1096 first year medical students (616 Dutch and 480 Swedish) with the validated Nijmegen Gender Awareness in Medicine Scale (N-GAMS). The response rate was 94% in the Netherlands and 93% in Sweden. Univariate analysis of variance (ANOVA) was used to compare the scores between Dutch and Swedish male and female students. Linear regressions were used to analyze the importance of the background variables.

**Results:**

There were significant differences in attitudes to gender between Dutch and Swedish students. The Swedish students expressed less stereotypical thinking about patients and doctors and the Dutch were more sensitive to gender differences. The students' sex mattered for gender stereotyping, with male students in both countries agreeing more with stereotypical statements. Students' age, father's birth country and mother's education level had some impact on the outcome.

**Conclusions:**

There are differences between cultures as well as between men and women in gender awareness that need to be considered when implementing gender in medical education.

This study suggests that to arouse the students' interest in gender issues and make them aware of the significance of gender in medical work, the examples used in discussions need to be relevant and challenging in the context of the specific country. Due to different levels of knowledge and different attitudes within the student population it is important to create a climate for dialogue where students feel permitted to disclose their ideas and attitudes in order to become aware of what these are as well as their possible consequences on interaction and decision-making in medical work.

## Background

Differences between men and women regarding biology, living conditions, behaviours and risk-taking are important causes of gender disparities in health and illness [1,2]. The impact of gender is not limited to reproduction-related disorders but also relevant in conditions such as cardiovascular disease, psychiatric disorders and cancer. Moreover, gender has an impact on medical communication, influencing patients' symptom presentations [3,4] as well as doctors' conduct and interpretations of patients' complaints and signs [5,6]. For example, physicians are more likely to interpret men's symptoms as organic and women's as psychosocial [5,7], and female patients are less often considered for referral or further investigation than men with similar symptoms, which raises questions of unequal care and gender bias [6,8,9].

As the importance of gender in health care has become established knowledge, attention has been paid to the implementation of gender issues in medical schools. Research reports and political documents are pointing towards the need for gender perspectives in medical education in order to ascertain good and appropriate health care for both men and women and increase future doctors' gender awareness [2,10]. Gender awareness means that physicians have the knowledge and ability to recognize and incorporate gender as an essential determinant of health and illness into their daily practice [11]. Gender awareness also denotes being aware that stereotyped assumptions and beliefs about men's and women's behaviour, skills, and needs are widespread in society and reframing people's thinking and perceptions. Since gender-stereotypical thinking has the inherent risk of thwarting and biasing medical assessments, gender awareness implies reflecting on one's own attitudes and preconceptions about men and women, patients as well as doctors and other staff [9,12].

In most countries and medical schools the process of including gender aspects in medical curricula has started only recently. Reports from Canada, the Netherlands, Sweden and Australia describe the achievements and work undertaken, for example in pedagogy and the development of databases with paper-cases that incorporate gender aspects [11,13-15]. The implementation of gender is not always a smooth process; it is sometimes met with doubts and scepticism [15]. In a Swedish study, where a majority of the medical students considered gender to be an important topic with relevance, another group of students were hesitant and some even expressed reluctant and negative views about the subject [16]. To achieve successful implementation, it is necessary to investigate attitudes towards and values about gender issues [12,17]. Recently the Nijmegen Gender Awareness Scale in Medicine, (N-GAMS) for measuring medical students' attitudes and values concerning gender was developed and validated in the Netherlands [11]. The scale was suggested as a tool for making a baseline assessment of gender awareness when implementing gender perspectives, as well as an evaluative assessment after the integration of gender. When used in a cross-sectional study in the Netherlands, N-GAMS showed that male medical students held stronger gender stereotypes than their female peers [11]. In the present paper N-GAMS is used to compare gender awareness in first-term Dutch and Swedish students.

The Netherlands and Sweden are two countries in the front row when it comes to implementing gender within medical education. The need for gender perspectives in medical curricula has been acknowledged at governmental level in both countries. The Dutch Ministry of Health initiated a nationwide project for implementing gender issues in Dutch medical schools in 2002; the project had its centre at Radboud University, Nijmegen Medical Centre [11]. In Sweden, the government has initiated several assessments of education about gender in medical schools and this has had effects on local university policies. In 2001, Umeå University medical school decided to mainstream gender perspectives into the medical curriculum, and a committee was set up to lead this work [14].

Even though the Netherlands and Sweden are both wealthy European countries, gender relations in the two countries show differences. The Dutch two-third earner model implies that men work full-time while women work part-time and take care of their own children or grandchildren [18]. Dutch women's labour market participation started rather late in the 1980s, and among all industrialized countries the Netherlands ranks first in the list for part-time work among women [19,20]. In Sweden, almost as many women as men (80%) participate in paid work and every other woman of working age works full-time [21]. Day-care facilities and support for Swedish parents in terms of pregnancy and parental leave are well developed and widely used since the 1960s [20].

Feminist research argues that social policies in different countries can be seen as representations of gender values and ideology on a structural level [22]. Social policies may affect individuals' behaviour, for example how they choose to study, work, or organize their private lives, and, in turn, people's behaviours have an impact on general assumptions and expectations of men and women, as well as of how gender differences are perceived [23]. For medical education this means that there might be country differences in students' attitudes to and perceptions of gender. To explore this, the aim of this study is to compare attitudes to gender and gender stereotyping among Dutch and Swedish male and female medical students.

## Methods

This study had a cross-sectional design and data were collected by a questionnaire. First-year medical students at Umeå University in Sweden, from autumn 2006 to spring 2009 (n = 516, 46% men), and at Radboud University Nijmegen Medical Centre in the Netherlands, during 2006-2007 (n = 657, 32% men), were invited to participate. The students were informed about the study when attending an ordinary lecture during the first week in medical school. Participation was voluntary and those who agreed to participate stayed on after the lecture to anonymously answer the questionnaire. The Ethical Committee at Umeå University has approved this study.

### Questionnaire

The questionnaire measured gender awareness by means of the Nijmegen Gender Awareness in Medicine Scale (N-GAMS), previously elaborated in the Netherlands by way of exploratory factor analyses [11]. Reliability and validity were established and in its final form N-GAMS contains 32 statements answered by a 5-point Likert-scale (ranking from 1 = totally disagree to 5 = totally agree). It measures three dimensions of gender awareness: Gender Sensitivity (GS), focusing on students' attitudes towards gender concerns in health care; Gender Role Ideology towards Patients (GRI-P), measuring gender-stereotypical thinking about patients; and Gender Role Ideology towards Doctors (GRI-D), measuring gender-stereotypical thinking about doctors (Table 1). A high score on the GS scale implies more gender sensitivity. On the GRI-P and GRI-D subscales a higher score indicates agreeing more with gender stereotypes. In the present study one item was added to the GRI-D subscale to obtain an equal number of statements about male and female doctors.

**Table 1:** The N-GAMS scale, also showing items excluded*, scored in reverse_R and added to original scale_A

GS, Gender sensitivity (excluded items*, items scored in reverse_R)	
GS1_R	addressing differences between men and women creates inequity in health care*
GS2	physicians' knowledge of gender differences in illness and health increases quality of care*
GS3_R	physicians should only address biological differences between men and women
GS4_R	in non-sex-specific health disorders the sex/gender of the patient is irrelevant
GS5_R	a physician should confine as much as possible to biomedical aspects of health complaints of men and women
GS6_R	physicians do not need to know what happens in the lives of men and women to be able to deliver medical care*
GS7_R	differences between male and female physicians are too small to be relevant
GS8_R	especially because men and women are different, physicians should treat everybody the same
GS9_R	physicians who address gender differences are not dealing with the important issues
GS10_R	in communicating with patients it does not matter to a physician whether the patients are men or women
GS11_R	in communicating with patients it does not matter whether the physician is a man or a woman
GS12_R	differences between male and female patients are so small that physicians can hardly take them into account
GS13	for effective treatment, physicians should address gender differences in etiology and consequences of disease*
GS14_R	it is not necessary to consider gender differences in presentation of complaints*

GRIP, Gender role ideology towards patients (excluded item*)	

GRIP1	male patients better understand physicians' measures than female patients
GRIP2	female patients compared to male patients have unreasonable expectations of physicians
GRIP3	women more frequently than men want to discuss problems with physicians that do not belong in the consultation room
GRIP4	women expect too much emotional support from physicians
GRIP5	male patients are less demanding than female patients
GRIP6	women are larger consumers of health care than is actually needed
GRIP7	men do not go to a physician for harmless health problems
GRIP8	medically unexplained symptoms develop in women because they lament too much about their health
GRIP9	female patients complain about their health because they need more attention than male patients
GRIP10	it is easier to find causes of health complaints in men because men communicate in a direct way
GRIP11	men appeal to health care more often with problems they should have prevented*

GRID, Gender role ideology towards doctors (excluded item*, item added _A)	

GRID1	male physicians put too much emphasis on technical aspects of medicine compared to female physicians
GRID2	female physicians extend their consultations too much compared to male physicians
GRID3	male physicians are more efficient than female physicians
GRID4	female physicians are more empathic than male physicians
GRID5	female physicians needlessly take into account how a patient experiences disease
GRID6	male physicians are better able to deal with the work than female physicians*
GRID7	female physicians are too emotionally involved with their patients
GRID8_A	compared to female physicians, male physicians are too hurried in their consultations

*items removed during factor analysis due to low factor loading.

_R items scored in reverse, i.e. the more you agree, the lower your gender sensitivity score.

_A item added to the original N-GAMS scale to achieve an equal number of statements about male and female doctors.

The questionnaire also contained questions about the students' and their parents' socio-cultural background, i.e., students' sex, age, country of birth, civil status and whether they had children or not, and parents' country of birth, educational level and working time. The educational level of the parents was divided into three categories: "primary" when the highest education was primary school, "intermediate" when the highest education was secondary or intermediate secondary school, and "higher" for higher secondary school or university. The parents' working time was reported as "no paid work", "part-time" (< 36 hrs/week) or "full-time" (≥ 36 hrs/week).

### Validation of the scale

To check the N-GAMS scale for our sample including both Dutch and Swedish medical students, exploratory factor analyses were performed. The three-factor structure was confirmed, the new item loaded as expected together with the other GRI-D statements, but seven other statements were excluded due to low factor loadings (< .4) or cross-loading (Table 1). Factor analysis was repeated without the excluded items and all factor loadings were >.437. The three factors extracted explained 42.5% of the variance (GS 9.6%, GRI-P 28.4% and GRI-D 4.5%). The reliability scores of the subscales with Cronbach's α were: GS-scale α = .76 (9 items), GRI-P scale α = .89 (10 items) and GRI-D scale α = .89 (7 items). GRI-D and GRI-P were significantly correlated (r = .657, p < 0.000) supporting a common ground for gender stereotypes towards physicians and patients. However, extracting three components also indicated a distinction between both subscales.

### Analysis

Univariate analysis of variance (ANOVA) was used to compare results from multiple groups. The N-GAMS scores between Dutch and Swedish male and female students was compared and interaction effects between country of study and student's sex was analysed.

To analyse the relationship between the socio-cultural background variables and outcome on subscales of the N-GAMS we performed linear regressions since the outcome variables are interval variables. We used a model with students' and parents' background variables as independent variables and the outcome on GS, GRI-P and GRI-D as dependent variables. Since ANOVA showed differences between the Dutch and Swedish students we stratified our analyses by country. All statistical analyses of the data were performed in SPSS 17.0.

## Results

In total 1096 (616 Dutch and 480 Swedish) students participated in the study, a response rate of 94% in the Netherlands and 93% in Sweden (Table 2). The response rate among men and women was similar in each country (not shown in table).

**Table 2:** Dutch and Swedish medical students' characteristics

Variable	Categories	*The Netherlands*	*Sweden*	P
Age, mean (SD)		18.8 (1.70)	22.7 (3.70)	< 0.001†
Sex, % (n)	Male	30.5 (188)	46.0 (221)	< 0.001^Ω^
	Female	69.5 (428)	54.0 (259)	
Sexual orientation	Heterosexual	98.2 (605)	94.6 (452)	0.001 ^Ω^
	Other	1.8 (11)	5.4 (26)	
Civil status	Single	97.1 (598)	78.3 (376)	< 0.001 ^Ω^
	Cohabiting/married	2.9 (18)	21.7 (104)	
Children	Yes	0 (0)	4.2 (20)	< 0.001 ^Ω^
	No	100 (612)	95.8 (457)	
Birth country student	Country of study	94.6 (575)	95.8 (459)	0.341 ^Ω^
	Other	5.4 (33)	4.2 (20)	
Birth country mother	Country of study	90.3 (543)	91.9 (440)	0.389 ^Ω^
	Other	9.7 (58)	8.1 (39)	
Birth country father	Country of study	89.1 (541)	91.0 (436)	0.302 ^Ω^
	Other	10.9 (66)	9.0 (43)	
Highest education mother	Primary	1.9 (12)	2.3 (11)	< 0.001 ^Ω^
	Intermediate	45.9 (283)	15.9 (76)	
	Higher	52.1 (321)	81.8 (392)	
Highest education father	Primary	1.1 (7)	5.0 (24)	< 0.001 ^Ω^
	Intermediate	30.6 (187)	19.5 (93)	
	Higher	68.2 (417)	75.5 (360)	
Mother's working status	No paid work	18.2 (111)	10.0 (47)	< 0.001 ^Ω^
	Part-time	66.7 (407)	21.1 (99)	
	Full-time	15.1 (92)	68.9 (324)	
Father's working status	No paid work	6.5 (39)	9.0 (42)	0.067 ^Ω^
	Part-time	8.8 (53)	5.8 (27)	
	Full-time	84.6 (507)	85.2 (396)	

Total		56.2 (616)	43.8 (480)	

Age presented as means (SD), other factors % (n).

Percentages are valid percent, missing values excluded, therefore the sum of n is not always the sum of participants. Most missing values concern father's working status (2.9%, n = 32).

†p value calculated using t-test

^Ω ^p values calculated using Chi-Square, level of significance p < 0.05

The students' age ranged from 16 to 50 years (M = 21), the Swedish students were older than the Dutch (M = 23 years versus M = 19 years) and the age span was larger in Sweden. There were more female students in both samples but the preponderance of women was considerably more pronounced in the Netherlands, where about 70% of the participants (and eligible students) were women. It was more common that Swedish students were cohabiting or married, had children, or that they reported a sexual orientation other than heterosexual, compared to the Dutch peers. In both Sweden and the Netherlands the proportion of students with a mother or father born abroad was small, and in both countries the non-native parents had their origin in many different countries in Europe, Asia, North America, South America and Africa (not shown in table). The parents' level of education was higher in Sweden than in the Netherlands and the difference was especially large between the Swedish and Dutch mothers. Concerning parents' working status, a large difference was also found between the mothers. A majority of the Swedish students had full-time working mothers but the most common among the Dutch was that mothers worked part-time.

Since none of the Dutch students had children this variable was excluded in further analyses.

The outcome on N-GAMS showed significant differences between Dutch and Swedish students on all three subscales when compared by ANOVA (Table 3). The Dutch students reported higher gender sensitivity, i.e., they had higher scores on the GS subscale. Dutch students also had a higher score than the Swedish students on the GRI-P and GRI-D subscales, indicating that Dutch students held more gender-stereotypical attitudes towards both doctors and patients. When comparing N-GAMS scores for male and female students, a significant difference was found on the GRI-P scale, where the male students demonstrated more stereotypical attitudes than female students. No significant interaction effects were found. The mean values for the subscales showed that Swedish female students had the lowest values on GRI-P, i.e., they expressed the least stereotypical thinking towards patients. Thereafter came the Swedish male students, Dutch female students and finally the Dutch male students. The mean values for the GRI-D subscale showed the same pattern. The Swedish female students agreed the least and the Dutch male students agreed the most with stereotypical thinking about male and female doctors.

**Table 3:** Comparison of results on the N-GAMS subscales

	**The Netherlands**		**Sweden**		Main effect country			Main effect sex			Interaction effect		
	**Male** **(n = 188)** **Mean** **(SD)**	**Female** **(n = 428)** **Mean** **(SD)**	**Male** **(n = 221)** **Mean (SD)**	**Female** **(n = 259)** **Mean (SD)**	**F (df)**	**sig**	**η^2^**	**F**	**sig**	**η^2^**	**F**	**sig**	**η^2^**

GS	3.43 (0.58)	3.43 (0.53)	3.30 (0.66)	3.37 (0.64)	5.87 (3, 1054)	0.016	0.006		NS			NS	
GRIP	2.52 (0.59)	2.27 (0.51)	1.96 (0.74)	1.70 (0.59)	221.34 (3, 1076)	< 0.001	0.171	43.97	< 0.001	0.039		NS	
GRID	2.44 (0.60)	2.43 (0.55)	2.07 (0.76)	2.00 (0.72)	93.96 (3, 1073)	< 0.001	0.081		NS			NS	

Univariate analysis of variance used, significant results shown.

GS: gender sensitivity, GRI-P: gender role ideology towards patients, GRI-D: gender role ideology towards doctors.

N represents total number of participants, missing values excluded: GS 3.8%, n = 38, GRI-P 1.5%, n = 16, GRI-D 1.7%, n = 19.

When exploring whether the socio-cultural background variables were related to outcome on GS, GRI-P and GRI-D, linear regressions stratified by country were used. Within this model (Table 4) age was important for GS in both countries, with older students scoring higher, i.e. being more gender sensitive. Father's country of birth had an impact on GS in the Dutch sample, i.e., Dutch students with non-native fathers were less gender-sensitive. The students' sex was significant for the outcome on GRI-P in both countries, but explained this score to a greater extent in the Netherlands than in Sweden. In Sweden, older students also expressed less stereotypical thinking about patients. Mothers' education was the largest contributor in our model among the Swedish students, and students with intermediate- or high-educated mothers disagreed more with stereotypical thinking about patients and doctors than did students with low-educated mothers.

**Table 4:** Relationship between background variables and outcome on N-GAMS subscales through linear regression stratified by country

Variable	Categories	*GS*		*GRIP*		*GRID*	
				
		*Dutch*	*Swedish*	*Dutch*	*Swedish*	*Dutch*	*Swedish*
Sex	Man (ref)						
	Woman	-0.013	0.077	-0.217***	-0.189***	0.005	-0.043
Age		0.158***	0.186**	-0.025	-0.190**	0.040	-0.084
Birth country	Country of study (ref)						
	Other	0.059	0.008	0.001	0.052	-0.025	0.084
Sexuality	Heterosexual (ref)						
	Other	-0.007	0.095	0.003	-0.067	-0.028	0.015
Civil status	Single (ref)						
	Cohabiting/Married	0.046	-0.023	-0.042	0.029	-0.060	0.002
Birth country father	Country of study (ref)						
	Other	-0.166*	-0.094	0.045	0.008	0.124	-0.013
Birth country mother	Country of study (ref)						
	Other	-0.003	0.053	0.025	-0.063	-0.068	-0.020
Education father	Low (ref)						
	Intermediate	-0.080	0.098	-0.254	0.064	0.084	0.095
	High	-0.023	0.172	-0.290	0.039	0.032	0.087
Education Mother	Low (ref)						
	Intermediate	0.018	-0.111	0.305	-0.332**	0.231	-0.261*
	High	0.102	-0.068	0.289	-0.319*	0.179	-0.231
Working status father	No work (ref)						
	Part-time	-0.025	0.041	0.069	-0.033	0.115	-0.068
	Full-time	0.031	0.092	0.107	-0.017	0.150*	-0.048
status mother	No work (ref)						
	Part-time	0.024	-0.115	-0.023	-0.091	0.026	-0.090
	Full-time	-0.026	-0.130	0.029	-0.073	0.029	-0.071

R^2 ^total		0.036	0.031	0.041	0.052	0.005	-0.009

GS: gender sensitivity, GRI-P: gender role ideology towards patients, GRI-D: gender role ideology towards doctors.

Numbers shown are standardized regression coefficient (Beta).

*p < 0.05

** p < 0.01

***p < 0.001

However, as indicated by R^2 ^in Table 4 (degree of explanation), in this model the socio-cultural variables did not contribute much to the outcome on N-GAMS.

## Discussion

The Dutch and Swedish medical students differed in gender awareness and showed significant differences in outcome on all three N-GAMS subscales. Dutch students reported higher gender sensitivity but they also expressed more gender-stereotypical attitudes towards patients and doctors. There were differences between men and women in stereotypical thinking, and in both countries female students disagreed more with stereotypical statements about patients. The pattern was that Swedish female students expressed the least stereotypical thinking, followed by Swedish male students, Dutch female students and finally the Dutch male students. The students' age had some impact on outcome in both countries, father's country of birth was related to gender sensitivity among the Dutch students, and mother's education was related to stereotypical thinking among the Swedish students.

### On method

Data for this study were collected during the students' first week in medical school, implying that the results mirror the gender awareness the students had when entering their studies. The large sample and high response rate was a strength.

N-GAMS was carefully elaborated and validated in a Dutch context [11], but our factor analysis showed that N-GAMS could be translated and used also among Swedish medical students. Factor structure was the same and the loadings similar in the Swedish and the Dutch samples. The items that needed to be excluded from the subscales had low loading in both samples. From GRI-P and GRI-D, one item from each subscale needed to be excluded (Table 1). The item added to the GRI-D subscale loaded as expected and seemed to add something to the scale. The GS subscale needed the largest revision, with five items excluded due to low factor loading. The remaining nine items all concern whether differences between men and women are relevant to consider in clinical work. In fact, "sensitivity for gender differences" might be a more appropriate name than "gender sensitivity" for this subscale.

Using a scale as a tool for exploring gender attitudes and stereotypes obviously has both strengths and limitations. The N-GAMS scale was suggested to be used for research purposes and to evaluate gender awareness raising courses and one obvious strength with using such an instrument is that a large number of medical students could be included in the study. We also found that N-GAMS could be translated and used outside the Netherlands, meaning that the instrument makes it possible to compare medical students in different countries and cultural settings. However, the methods needs certain reflection; when understanding the differences we found between Dutch and Swedish students the background variables used in the questionnaire do not in themselves explain the results. Still, the significant differences in students' characteristics (Table 2) provide clues to the context of the medical students' attitudes and awareness. While the N-GAMS scale could be used for example as a baseline assessment to evaluate gender awareness raising courses, or to make a comparison of a large amount students, qualitative research might give further depth and provide more explanations through highlighting the social discourses and reasoning that lies behind the students results and the difference between the Netherlands and Sweden.

### On Results

The fact that the students in Nijmegen compared to the students in Umeå were more sensitive to gender differences and also more gender stereotyping of patients and doctors might have different explanations. Since the students came from all over Sweden and the Netherlands and answered the questionaire during their first days of medical school it is reasonable to believe that the differences reflect national differences rather than University differences. As outlined in the introduction, Sweden and the Netherlands are both welfare states, but the Netherlands has less far-reaching gender equality legislation and a more obviously gendered distribution of chores and duties than Sweden [19,20,23]. Childcare and household work are the responsibilities of Dutch women who to a large extent work part-time, while most Dutch men are the main breadwinners and work full-time. In Sweden a majority of both women and men work full-time while the children are taken care of at day-care centres [21]. Conditions in society and people's behaviour have an impact on people's attitudes, expectations and values [22], and inasmuch as Dutch students see more differences between men and women in everyday activities it seems reasonable that they perceive gender differences as more relevant and also score higher for GS than the Swedish students do.

While the statements in the GS subscale consider the importance of biological and communicative gender differences for the clinical work, the statements in the GRIP and GRID subscales contain rather outspoken and evaluative statements about how male and female patients and doctors *are*, for example that patients or doctors of one gender are "better", "too much" or "less", compared to the opposite gender (Table 1). Agreeing with the statements implies believing in gender differences, but also accepting a hierarchy in the differences - the features of one gender are described as being superior to and more desirable than the features of the other. In line with common gender stereotypes in society [24,25], the statements describe men as more competent, effective, instrumental and trustworthy than women, who are seen as more emotional, worried and in need of more attention and time to talk. That the Swedish students disagreed more than the Dutch with gender-stereotypical statements was not so surprising. The dominant political discourse about gender in Sweden proclaims that women and men *are *equal and *should be treated *equally [26]. These social norms reasonably influence not only students' values but also what they consider to be suitable and political correct answers in an enquiry.

Although communal conceptions about men and women underpin attitudes towards both patients and doctors, the students' gender stereotyping of patients was more pronounced than their stereotyping towards doctors. Previous research shows that people's self-reports of their instrumental and communal traits are usually less gender-stereotypical than are their estimates of the "typical person" [24,27]. Perhaps students in the first term already identify with the group of "doctors", and knowing the diversity of their own features they avoid judging doctors according to preconceptions about typical male and female behaviours?

The finding that male students held stronger gender stereotypes towards patients than their female peers is consistent with the previous Dutch study (using N-GAMS) of medical students [11], and with other research comparing gender-stereotypical attitudes in men and women [24,25]. In the present study both male and female students scored in the direction of disagreeing with stereotypes, but the female students stated more clearly that they disagree. The reason that men admit more to gender stereotypes could be that such stereotypes are in general more positive towards men, which is also true for the statements on the GRI-P subscale [25].

Proportions of women in full-time work and educational level in both men and women are often used as indicators of gender equality in a country [19,20,23]. We therefore expected that these background variables would contribute to explaining the differences in gender awareness between the medical students in the Netherlands and Sweden. However, no such clear pattern emerged. How can that be understood?

It is well described in research that prevailing norms and beliefs about gender in a society are created and maintained by multiple, complementary processes acting simultaneously and at different levels of analysis, e.g., individual, societal, political, normative and cultural levels [22,24,25]. Changes on a single level will not be sufficient to eliminate or change the gendered norm patterns or attitudes. For instance, even if political decisions aiming at gender equality have had an impact on women's career choices as well as on men's participation in traditional female tasks [19,20], studies on gender stereotypes show that the core structure of beliefs about typical men and typical women have been largely unchanged for decades [24,25,27]. Gender stereotypes have a high momentum of inertia and change slowly. Transferring this reasoning to our study, Swedish and Dutch students with parents acting in contrast to traditional gender roles are still living in societies where gendered segregation of duties is a prevailing pattern and where cultural beliefs about gender are exposed in everyday talk as well as in newspapers, books, films, and other media. Thus it becomes hard to relate the differences in attitudes between the Dutch and Swedish students to one or the other of the characteristics used as background factors. Moreover, within each country of study, the variation among the students in socio-cultural background is small and a majority of the students have similar background characteristics (Table 2). When the countries are separated and the background variables analysed in linear regression, most students are grouped in the same categories, which would explain that even the model including all background variables does not explain more of the variation in outcome on N-GAMS (Table 4). Still, our study indicates that mothers' educational level is important for gender attitudes.

### Implications for education

It is important to know the students' attitudes as a take-off point in the implementation of gender in the medical curricula [12,17]. Different attitudes between the Dutch and Swedish students informs us that medical education has to take cultural and societal differences between countries into account, and even when comparing two welfare western states there are differences in values and preconceptions about gender. Inasmuch as the goal in education about gender is to make students interested and aware of the significance of gender in medical work, the examples used in discussions need to be trustworthy, relevant and challenging in the context of the specific country.

In our study the male students agreed more with gender-stereotypical statements about patients than their female peers, which is in line with previous research showing that men to a larger extent than women hold on to gender-stereotypical ideas [11,24]. Earlier studies have shown that male students are less knowledgeable about gender issues and also more sceptical about the implementation of gender in education than female students [15,16]. We suggest that these phenomena are related. Lack of insight and knowledge implies difficulties in understanding the relevance of gender in medical work, and not understanding the relevance makes you less interested in, and more sceptical about, education on gender. Furthermore, lack of interest means that you are less motivated to reflect on your own attitudes and values concerning gender and you are thus more subject to stereotyped preconceptions about men's and women's needs, wishes, and behaviour. Is it possible to break through this circular process? Yes, to some extent at least. Previous research claims that students become more positive and interested as they learn more and get used to discussing gender [15,16]. However, to the best of our knowledge, there is a lack of research about whether tendencies to make stereotyped assessments of patients decrease as a result of the implementation of gender aspects in education.

Since gender-stereotypical assessments might lead to bias and misjudgements of patients, as well as misunderstandings in collegial cooperation, it is important to find out how to help students to reduce judgments that are grounded in stereotypes [9,12]. Reflecting about one's own reactions to patients in clinical situations might be a tool that helps students to see their own attitudes and the consequences these might have for medical treatment. Such reflections can proceed from the students' own experiences from e.g. clinical training in hospitals and primary care, in prefabricated paper-cases, or in symptom presentations from simulated patients.

Our experiences from gender education indicate that, parallel to the importance of good examples to discuss and reflect on, it is crucial to create a climate for dialogue where students feel permitted to disclose ideas and attitudes, including such ideas as are not "politically correct" in a specific context. If problematic ideas and reactions are not disclosed, the students' chances to catch sight of their own attitudes and values, and to discuss them, will be small and as a result the education will have less impact. On the other hand, when a student realizes that her/his own, or other students' or staff members', values and preconceptions might lead to problems for patients, then s/he usually becomes curious and interested.

Further research about the effects of gender perspectives in education on students' attitudes and behaviour need to focus the gender topics included as well as the education methods used.

## Conclusions

This study shows that the Nijmegen Gender Awareness Scale in Medicine (N-GAMS) can be used for international comparisons of medical students' attitudes and values concerning gender.

There are differences between cultures as well as between men and women in gender awareness that need to be considered when implementing gender in medical education.

Based on these results we suggest that to arouse the medical students' interest in gender issues and make them aware of the significance of gender in medical work it is crucial that the examples used in discussions are trustworthy, relevant and challenging in the context of the specific country. Due to different levels of knowledge and different attitudes within the student population it is important not only with good examples to discuss and reflect on, but also to create a climate for dialogue where students feel permitted to disclose their ideas and attitudes in order to become aware of what these are as well as their possible consequences for their future profession.

## Competing interests

The authors declare that they have no competing interests.

## Authors' contributions

All five authors have made contributions to the study design, acquisition and interpretation of data. TLJ and KH was responsible the Dutch and Swedish data respectively. JA was responsible for the coordination of data and performed the statistical analysis. PV developed and validated the N-GAMS scale and made substantial contributions to the analysis. JA drafted the manuscript and EJ and KH assisted in writing the manuscript. All authors have been involved in revising the manuscript critically for important intellectual content and have read and approved the final manuscript.

## Pre-publication history

The pre-publication history for this paper can be accessed here:

http://www.biomedcentral.com/1472-6920/12/3/prepub
